# A high-resolution binocular video-oculography system: assessment of pupillary light reflex and detection of an early incomplete blink and an upward eye movement

**DOI:** 10.1186/s12938-015-0016-6

**Published:** 2015-03-13

**Authors:** Julián Espinosa, Ana Belén Roig, Jorge Pérez, David Mas

**Affiliations:** Institute of Physics Applied to the Sciences and Technologies, University of Alicante, Alicante, Spain; Department of Optics, Pharmacology and Anatomy, University of Alicante, Alicante, Spain

**Keywords:** Binocular measurement, Pupillary light reflex, Early incomplete blink, Bell’s phenomenon

## Abstract

**Background:**

The pupillary light reflex characterizes the direct and consensual response of the eye to the perceived brightness of a stimulus. It has been used as indicator of both neurological and optic nerve pathologies. As with other eye reflexes, this reflex constitutes an almost instantaneous movement and is linked to activation of the same midbrain area. The latency of the pupillary light reflex is around 200 ms, although the literature also indicates that the fastest eye reflexes last 20 ms. Therefore, a system with sufficiently high spatial and temporal resolutions is required for accurate assessment. In this study, we analyzed the pupillary light reflex to determine whether any small discrepancy exists between the direct and consensual responses, and to ascertain whether any other eye reflex occurs before the pupillary light reflex.

**Methods:**

We constructed a binocular video-oculography system two high-speed cameras that simultaneously focused on both eyes. This was then employed to assess the direct and consensual responses of each eye using our own algorithm based on Circular Hough Transform to detect and track the pupil. Time parameters describing the pupillary light reflex were obtained from the radius time-variation. Eight healthy subjects (4 women, 4 men, aged 24–45) participated in this experiment.

**Results:**

Our system, which has a resolution of 15 microns and 4 ms, obtained time parameters describing the pupillary light reflex that were similar to those reported in previous studies, with no significant differences between direct and consensual reflexes. Moreover, it revealed an incomplete reflex blink and an upward eye movement at around 100 ms that may correspond to Bell’s phenomenon.

**Conclusions:**

Direct and consensual pupillary responses do not any significant temporal differences. The system and method described here could prove useful for further assessment of pupillary and blink reflexes. The resolution obtained revealed the existence reported here of an early incomplete blink and an upward eye movement.

**Electronic supplementary material:**

The online version of this article (doi:10.1186/s12938-015-0016-6) contains supplementary material, which is available to authorized users.

## Abstract (Spanish)

**Antecedentes:**

El análisis del reflejo pupilar a la luz caracteriza la respuesta directa y consensual del ojo al brillo percibido de un estímulo y puede utilizarse como indicador de patologías tanto neurológicas como en el nervio óptico. Al igual que otros reflejos, se trata de un movimiento rápido casi instantáneo y está ligado a la activación de la misma área del mesencéfalo. La latencia del reflejo pupilar es de alrededor de 200 ms, pero los reflejos oculares más rápidos son de sólo 20 ms. Por tanto, es necesario un sistema con suficiente resolución espacial y temporal para evaluarlos con precisión. En este trabajo, analizaremos si existe alguna discrepancia entre la respuesta directa y consensual en el reflejo pupilar a la luz así como si hay algún otro reflejo ocular anterior a éste.

**Métodos:**

Hemos construido un video oculógrafo binocular con dos cámaras de alta velocidad simultáneamente enfocando a ambos ojos y, con éste, evaluamos la respuesta directa y consensual de cada ojo. Para ello, hemos desarrollado un algoritmo que mediante la transformada circular de Hough detecta y rastrea la pupila y permite obtener los parámetros temporales que describen el reflejo pupilar a la luz a partir de la variación del radio en el tiempo. Ocho sujetos sanos (4 mujeres y 4 hombres de edades entre 24 y 45) participaron en este experimento.

**Resultados:**

Nuestra propuesta, que alcanza una resolución de 15 micras y 4 ms, ha proporcionado valores para los parámetros temporales que caracterizan el reflejo pupilar a la luz similares a los encontrados en trabajos previos y no se han hallado diferencias significativas entre los reflejos directo y consensual. Además, en torno a los 100 ms, encontramos un reflejo incompleto de parpadeo y un movimiento del ojo hacia arriba que puede corresponderse con el fenómeno de Bell.

**Conclusiones:**

Las respuestas pupilares directa y consensual no presentan ninguna diferencia temporal apreciable. El sistema y el método descritos podrían ser útiles para un análisis más a fondo de los reflejos pupilares y de parpadeo. Así, su capacidad resolutiva ha revelado un parpadeo temprano incompleto y un movimiento del ojo hacia arriba.

**Palabras clave:**

Medida binocular, reflejo pupilar a la luz, parpadeo temprano incompleto, fenómeno de Bell

## Background

The size of the eye’s pupil automatically determines the amount of light arriving to the retina [1-3], and this it plays an important role in the visual system. Pupil dynamics has been used as an indicator of optic nerve disease [4-6] and neurological injury [7-10], or to assess the circadian clock [11-13].

The pupillary light reflexes (PLR) characterizes the eye’s response to the perceived brightness of a stimulus, and has been classified in two types: the direct and the consensual response. The difference between these two responses lies in the stimulus: when the pupil contracts because it is receiving light, this is called the direct response, whereas if it contracts because the opposite eye is receiving light, this is called the consensual response. In humans, these two responses are thought to be identical [14,15], and any divergence between them is attributed to neurological pathology. Nevertheless, some studies have found that the amplitude of the consensual response is smaller than that of the direct one [16,17], and that it is sex-dependent [18].

The diameter of the pupil is regulated by two antagonistic muscles of the iris that are controlled through sympathetic and parasympathetic pathways [19]. The latter controls the sphincter responsible for the contraction of the pupil (miosis), whereas the sympathetic pathway controls dilation (mydriasis). The PLR may be linked to activation of the Superior Colliculus, a midbrain area also involved in preliminary visual processing and the control of eye movements such as orienting responses and saccades [20-22]. Saccades are produced as part of the normal process of vision in as little as 120 ms [23], or as a reaction to an unexpected stimulus. In this latter case, the response can be as fast as 90 ms, can lasts between 20–200 ms [24] and can be assessed using eye trackers, which have the capacity to measure up to 1000 Hz [25]. Analysis of saccades is directly related to tracking the line of sight, whereas assessment of the PLR basically involves recording pupil diameter and dynamics. Some parameters related to time characterization of the PLR in real eyes [26-29] include latency from the flash exposure to the start of contraction (T_1_), latency to smallest pupil size (T_2_) and latency to a plateau (T_3_). Usually, the start of contraction is defined as the time when the second time-derivative of radius evolution is at a minimum and T_3_, the end of the contraction process, is the instant after T_2_ when the second time-derivative peaks.

Pupillary measurement methods have varied from direct observation using rulers or circles, photographic techniques, and electronic pupillographs [30,31], to computerized pupillometry. In all cases, spatial and temporal resolutions are the most significant metrological parameters since they determine measurement precision.

Electronic pupillographs scan irises at a rate of 60 Hz, and have the capacity to detect pupillary responses of the order of 50 μm [32]. Despite having high spatial resolution, they are at the limit of temporal resolution for accurately registering fast eye movements, which last in the order of tens of milliseconds. Meanwhile, using computerized pupillometry, Hachol et al. [33] have attained linear spatial resolutions of up to 8 μm, but only recording at 90 Hz, which is also at the limit for accurately registering fast movements.

In addition, the use of eye trackers to assess the PLR has been reported in the literature. These devices are faster than classical pupillometry intruments; for example, the Eyelink II samples up to 500 Hz [13,21], another tracker from SensoMotoric also samples at 500 Hz [34] and the Eyelink 1000 reaches 1000 Hz [25]. In all cases, sampling rates are sufficiently high to detect reflexes in the order of milliseconds. However, those devices track eye movements rather than variations in pupil size, and spatial resolution must also be taken into account. In this respect, the from SensoMotoric device has a resolution of 200 microns, and although the Eyelink 1000 and Eyelink II devices provide a nominal pupil size resolution of 0.2 % of the pupil diameter, the measurements are affected by up to 10% due to optical distortion of the cornea and camera-related factors [35,36].

Consequently, none of the devices reported in literature fully meets the requirements for accurate measurement of pupillary dynamics in the order of tens of milliseconds. In principle, there should be no need to reach such a high temporal resolution since registered PLR latency is around 200 ms. However, if the PLR and saccades are related to activation of the Superior Colliculus, it is possible that an early response to light exists but has not yet been detected due to the limited resolution used to date. Therefore, it would be useful to develop a technique that attain such resolution.

Binocular registration of the PLR is required in clinical and research applications to assess direct and consensual pupil reactions. A binocular system has recently been proposed [37] based on a single camera that alternately records images of the left and right eyes. With a sampling rate up to 75 Hz and a resolution of more than 20 μm, this is sufficient to assess the PLR but again falls below the requirements to accurately register the fastest eye reflexes. Recently, some of the authors of this manuscript used a high-speed camera and image processing to analyze the eye blinking process [38,39] and fixational eye micromovements [40]. The main advantages of those experimental setups are that they have the capacity to sample data up to 1000 Hz. Using the same camera model, we constructed a binocular video-oculography system using two high-speed cameras which simultaneously focused on both eyes. This system was employed to determine whether there was any small discrepancy between the direct and consensual responses, and if saccades and the PLR are related as postulated earlier – to ascertain whether any other pupil reflex occurred below the 200 ms threshold.

The rest of the paper is structured as follows. First, in the method section, we describe the study participants, the experimental setup and the algorithm used in our work together with the subjects that participated in the study. Then, in the results section, we report the time parameters obtained for the PLR, which were similar to those described in previous studies, with no significant difference between direct and consensual reflexes. Nevertheless, our system, which yielded a resolution up to 15 microns and 500 Hz, revealed an upward eye movement that may correspond to Bell’s phenomenon [41,42] and an incomplete reflex blink [43] at around 100 ms, as we explain in the discussion. Therefore, the system and method described below could be useful for further assessment of these reflexes.

## Methods

### Experimental setup

**Figure 1:**
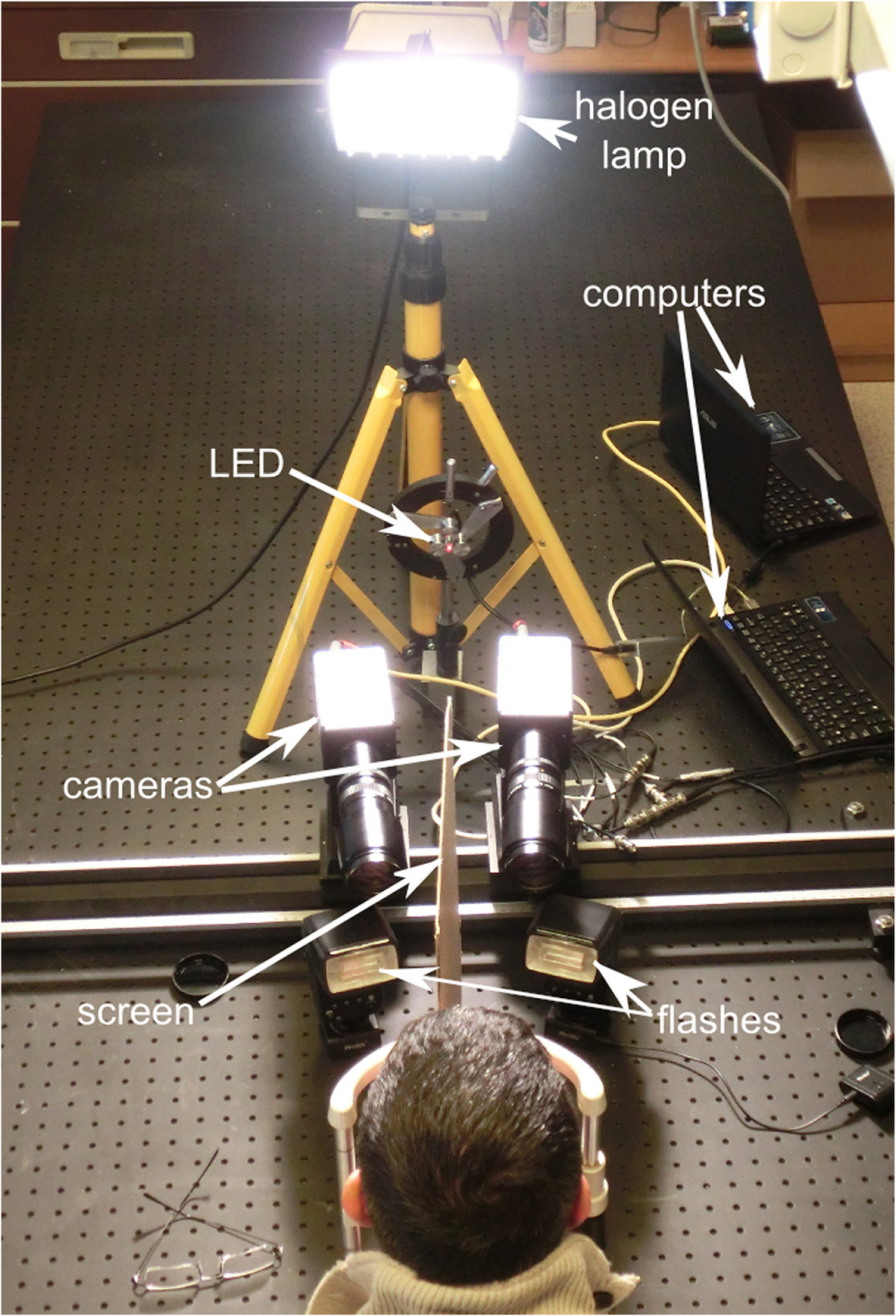
**Picture of the general experimental setup.** The image shows the arrangement of the elements used in the experiment.

Figure 1 shows the experimental setup used in this work. It consisted of a halogen projector that provided uniform illumination over the subject’s face (2170 ± 50 lux), a headrest with an opaque vertical screen that separated each eye’s filed of vision, two flash units and two digital video cameras (X-PRI AOS Technologies AC) which were synchronized via a wired connection following the manufacturer’s instructions. The cameras were positioned at a distance of 34 cm far from the headrest plane and acquired video sequences at a speed of 500 Hz and with a spatial resolution of 800 × 560px. Each camera focused on one of the subject’s eyes and was connected to a different computer. Thus, the system was capable of recording the pupillary dynamics of each eye separately but simultaneously.

The fixation point was a small red LED positioned 1.5 m from the plane of the headrest. Two flash units positioned at 34 cm from the same plane were used to elicit the PLR. Each of the flash units pointed at one eye and the screen prevented light passing the other side. When a flash was shot, illuminance reached a peak of 4100 ± 200 lux in the plane of the headrest.

One sequence of 4.20 seconds was registered for each of the subject’s eyes. Approximately one second after we started recording, and without giving the subject prior warning, we shot one of the flash units so that it only illuminated one eye. Since the light of the flash saturated the camera sensor, it was easy to identify the frame from when the flash was shot when we subsequently processed the sequences, and this frame was used to set the time to zero. The experiment was performed with the flash units stimulating each eye separately. This enabled us to assess both the direct and consensual responses of each eye, obtaining four sequences per subject.

Measurements were separated by a time interval of at least 15 minutes, and only one flash per session was applied. Subjects did not know which flash (left or right) would be shot. The recorded sequences (direct and consensual responses) were first stored in each camera and then transferred to laptops, which lasted around 15 minutes, to be processed off-line. If the sequence was not transferred, it was overwritten by a new recording.

### Pupil characterization

**Figure 2:**
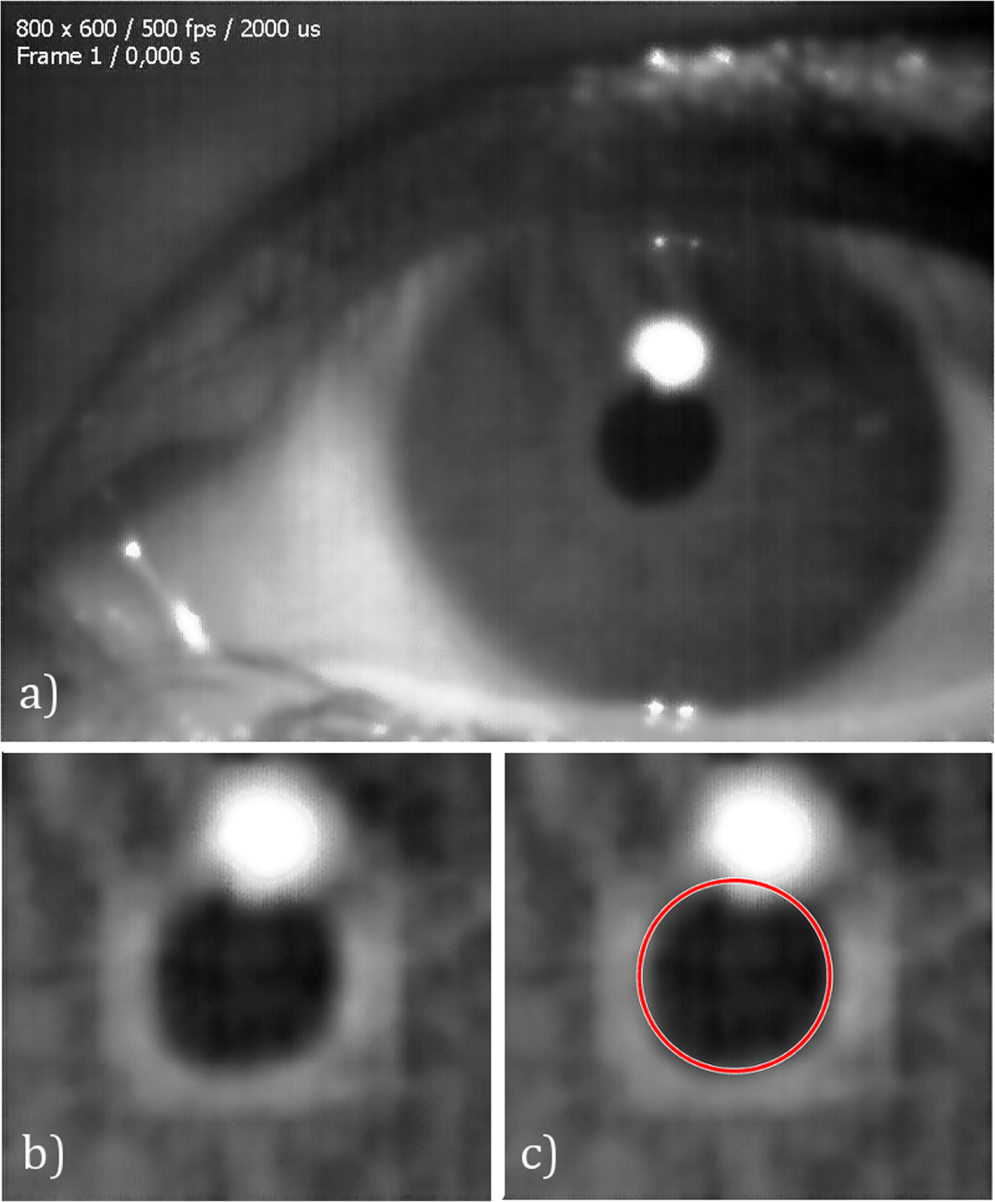
**Image processing phases. a)** Original frame, **b)** Wiener-filtered region of interest, **c)** circle drawn after determining its center using the Circular Hough Transform.

Recorded sequences were analyzed off-line using our own algorithm implemented in MATLAB in order to detect and track the changes in the pupil. In the first processing phase the pupil contour was determined in all frames. Starting with a gray scale image, Figure 2(a), we filtered it through pixel wise adaptive Wiener filtering [44] to remove noise, using local 9-by-9 px neighborhoods to estimate the local image mean and standard deviation. Then, we manually selected a region of interest (ROI) that contained the pupil, as shown in Figure 2(b), in order to reduce the processing time.

Noncircularities of real pupils are in the order of 0.02 [45], so we assumed a circular pupil. There are basically two ways of approaching pupil image processing reported in the literature; the Hough Transform [46,47] and best ellipse fitting [18,35-37,48]. We used a Circular Hough Transform (CHT) [49] based algorithm to detect circles in the images (Figure 2c), selecting this approach is used because of its robustness in the presence of noise, occlusion and varying illumination. The procedure consisted of two essential steps; first, high gradient foreground pixels were designated as being candidate pixels and were allowed to cast ‘votes’ in the accumulator array. The candidate pixels voted in a pattern around them that formed a full circle with a fixed radius. Thus, candidate pixels votes pertaining to an image circle tended to accumulate in the accumulator array bin corresponding to the circle’s center. Then, circle centers were estimated by detecting the peaks in the accumulator array.

### Sequence assessment

**Figure 3:**
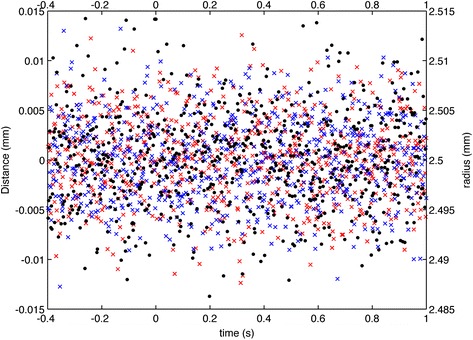
**Test of the method robustness using an artificial eye.** Time variations for pupil center coordinates and radii obtained for an artificial eye. Pupil center coordinates *x* and *y* are blue and red crosses, respectively (left axes). Black dots represent pupil radius (right axes). Flash is shot at t = 0 s.

First, we validated our experiment using with an artificial eye with 5 mm diameter pupil in order to assess the existence of any artifact due to hardware or software. The captured sequence was then processed to obtain the pupil radius and center coordinates in each frame. Measurement accuracy was defined as three times the standard deviation of these data. The result obtained from this experiment was 15 microns for both radii and center coordinates. These results are shown in Figure 3. The crosses represent the computed center coordinates, *x* in blue and *y* in red, referenced to the left axes and the black dots represent the computed pupil radii referenced to the right axes. The flash shot was used to set time to zero.

### Subjects

We asked eight healthy subjects (4 women, 4 men, aged 24–45) from among the staff at the Optics Department of the University of Alicante to participate in this experiment. Subjects were instructed and trained to avoid blinking. The experiment was conducted with the approval of the ethics committee of the University of Alicante, and in accordance with the declaration of Helsinki. All participants were informed about the nature and purpose of the study and they provided written informed consent to publish case details. All data used in the study were made anonymous.

## Results

**Figure 4:**
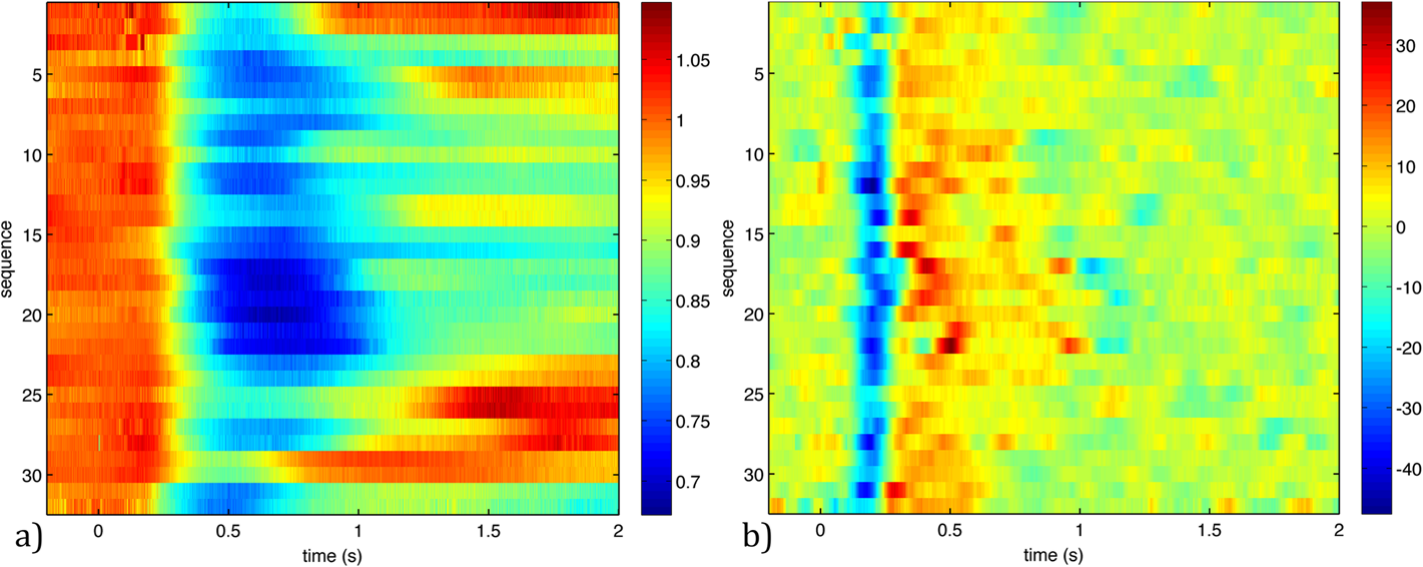
**Time variations in pupil radii. a)** Pupil radii relative to the radii at t = 0 s. **b)** Second time-derivative of the pupil radii evolution. Flash was shot at t = 0 s.

We analyzed the temporal evolution of the pupil radius according to the time parameters described earlier (T_1_, T_2_ and T_3_), studying 8 subjects, 2 eyes per subject, and the direct and consensual responses of each eye. We shot the flash once at each eye and recorded both responses each time, thus obtaining a total of 32 different measurements, i.e. 32 recorded and processed sequences. Figure 4a) shows the temporal evolution of pupil radius relative to the radius at t = 0 s (when the flash was shot), computed for all the sequences. As can be seen, that for all sequences the pupil radius abruptly decreased at around 0.2 s in all sequences. Then, at around 0.6 s, it reached a minimum of the 70% or 80% of the initial value and, finally, it increases its value.

Once we had processed each sequence and obtained the radii, we removed noise from the data using a Savitzky-Golay [50] filter and computed the second time-derivative. Figure 4b) shows values for the second time-derivative of the pupil radius for each measurement, where the color scale represents mm/s^2^. A band of minima clearly appears at around 0.2 seconds (T_1_), marking the start of the contraction.

Table 1 gives the average time parameters obtained for the PLR together with their standard deviation. Calculations were performed considering all eyes and distinguishing between direct and consensual reflexes. A comparison did not reveal any significant differences between them (p > 0.05). These results are in accordance with those reported in previous studies [26,28].

**Table 1:** **Time parameters describing the pupillary light reflex**

	**All**	**Direct**	**Consensual**	**p**-**value**
T_1_ (s)	0.199 ± 0.031	0.203 ± 0.021	0.195 ± 0.039	>0.05
T_2_ (s)	0.638 ± 0.083	0.642 ± 0.082	0.633 ± 0.086	>0.05
T_3_ (s)	1.562 ± 0.234	1.609 ± 0.215	1.516 ± 0.249	>0.05

Mean and standard deviation of time parameters of the PLR obtained from the sequences. A comparison between direct and consensual reflexes revealed no significant differences.

The time parameters describing the PLR determined by Fotiou et al. [26] are T_1_ = 0.17 ± 0.03 s, T_2_ = 0.64 ± 0.07 s and T_3_ = 1.86 ± 0.24 s; while those measured by Ferrari et al. [28] are T_1_ = 0.20 ± 0.078 s, T_2_ = 0.90 ± 0.147 s and T_3_ = 1.63 ± 0.559 s. We found that the value we obtained for T_1_ was in agreement with these. However, the value we obtained for T_2_ was similar to [26] but not to [28] while our value for T_3_ did not coincide with either. These disagreements may be due to slightly different parameter definitions or experimental characteristics. In fact, as Fotiou et al. [26] have stated, it is not possible to compare results between different studies because of differences in the systems used and the experimental conditions employed. First, the flash shot characteristics were different. Additionally, we measured under photopic conditions, so the initial pupil radius was smaller and this could have affected to the duration of the contraction.

**Figure 5:**
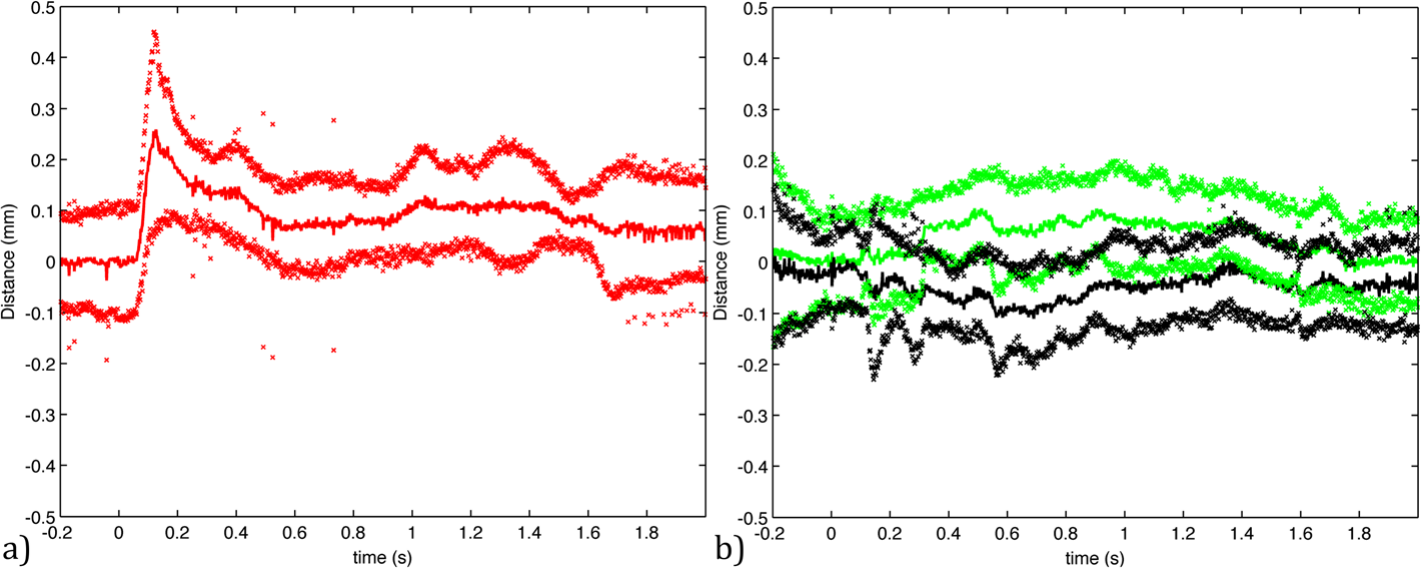
**Pupil center tracking.** Detrended mean evolutions in time of the pupil center: **a)** *y* coordinate and **b)** *x* coordinate, for the right (green) and left (black) eyes.

As regards pupil center tracking, in Figure 5a) shows the detrended mean temporal evolution of the *y* coordinate (red line), while in Figure 5b) shows the same for the *x* coordinate, distinguishing between the right (green line) and left (black line) eye. Crosses mark the confidence bounds (± standard deviation). No distinction is made between direct and consensual sequences because we did not found any significant differences. Figure 5a) clearly depicts a vertical movement that corresponded to an upward displacement of the eye of around 0.2 mm at 0.1 s after the flash (t = 0 s). Nevertheless, as can be seen in Figure 5b), no such reflex is apparent on the horizontal coordinate.

## Discussion

One possible explanation for this upward movement could be an incomplete reflex blink. Reflex blinks occur only in response to trigeminal, visual and acoustic stimuli [42]. The other two types of blinks are spontaneous ones, which occur in the absence of any evident stimulus, and voluntary ones, which are consciously willed by the subject. In these different types of blink, the eye movements accompany the eyelid movements and are probably due to the co-contraction of the superior and inferior rectus muscles [51]. An upward movement of the eye globes when blinking or when threatened is known as Bell’s phenomenon and is present in about 75% of the population [43]. Therefore, the registered movement could correspond to this phenomenon although, like the blink, it was incomplete.

**Figure 6:**
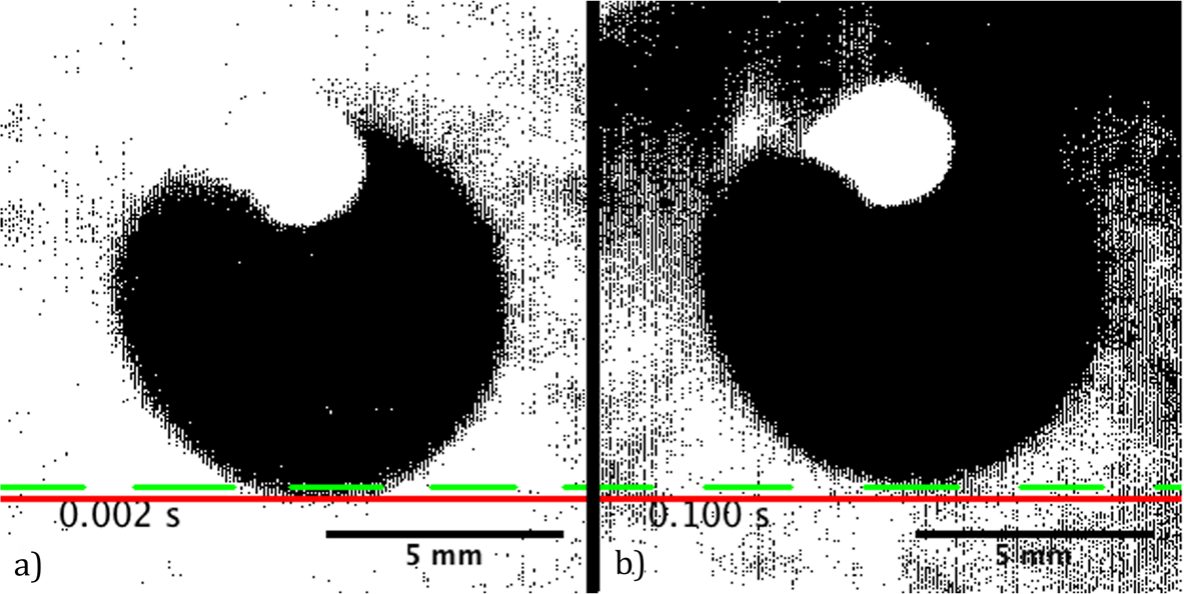
**Example of the upward pupil movement. a)** The first frame and **b)** the frame at 0.1 seconds after the flash shot, for one example pupil. The red line represents the initial position and the green dashed line represents the position at t = 0.1 s, clearly depicting the upward movement of the eye (Bell’s phenomenon) and the shadow of the upper eyelid (incomplete blink).

Additional file 1 shows the evolution of the pupil over 0.5 seconds after the flash shot, for an example subject. It has been properly thresholded to enhance the pupil over the background and depicts the upward movement of the pupil and the shadow of the upper eyelid (incomplete blink) at around 0.1 seconds. In Figure 6, we have extracted two frames from Additional file 1 to illustrate the upward eye movement that may be related to Bell’s phenomenon, and the incomplete blink.

The direction, magnitude and duration of Bell’s phenomenon are still controversial. Furthermore, different results have been reported depending on the type of blink assessed (spontaneous, voluntary or reflex) [52]. Francis and Loughead [53] assessed Bell’s phenomenon in 508 patients by elevating their upper eyelids and asking them to close their eyes. They classified movements of less than 4 mm as a small response. Note that our result shown in Figure 5 a) is a mean upper movement of less than 1 mm, a difference that may be due to the fact that we were not assessing a voluntary blink but a reflex one and this was not complete.

The proposed system could be useful for further assessment of Bell’s phenomenon, which helps in diagnosis and management of numerous systemic and ocular diseases (cerebral palsy, comatose patients, local orbital disease, etc.) and even reflects the maturation process of the brainstem and the extraocular muscles related to elevation [54]. Note that the method described here is non-invasive and there is no need to use magnetic search coils or electromyography recordings. Few studies in the literature have reported on measuring Bell’s phenomenon and, to the best of our knowledge, none of the methods described to date is non-invasive and performs the assessment simultaneously in both eyes.

## Conclusions

We have analyzed and characterized the pupillary light reflex under photopic condition in accordance with previous studies in the literature. A comparison of direct and consensual responses did not reveal any significant differences (p > 0.05). Although the PLR has been studied before, we thought that such a fast response should be accurately analyzed using high-speed imaging and the best possible spatial resolution. Working with a resolution of 4 ms and 15 microns, we have obtained evidence for the first time, to the best of our knowledge, of an incomplete blink reflex and Bell’s phenomenon prior to the PLR, which happened at around 100 ms.

## Additional file

Additional file 1:
**The video shows the evolution of the pupil during over 0.5 seconds after the flash shot, for an example subject.** It has been properly thresholded to enhance the pupil over the background and depicts the upward movement of the pupil and the shadow of the upper eyelid (incomplete blink) at around 0.1 seconds.
